# Introduction of the Utrecht Tasks for Attention in Toddlers Using Eye Tracking (UTATE): A Pilot Study

**DOI:** 10.3389/fpsyg.2016.00669

**Published:** 2016-05-06

**Authors:** Marjanneke de Jong, Marjolein Verhoeven, Ignace T. C. Hooge, Anneloes L. van Baar

**Affiliations:** ^1^Department of Child and Adolescent Studies, Utrecht UniversityUtrecht, Netherlands; ^2^Department of Experimental Psychology, Helmholtz Institute, Neuroscience and Cognition, Utrecht UniversityUtrecht, Netherlands

**Keywords:** orienting, alerting, executive attention, eye tracking, toddlers

## Abstract

Attention capacities underlie everyday functioning from an early age onwards. Little is known about attentional processes at toddler age. A feasible assessment of attention capacities at toddler age is needed to allow further study of attention development. In this study, a test battery is piloted that consists of four tasks which intend to measure the attention systems orienting, alerting, and executive attention: the Utrecht Tasks of Attention in Toddlers using Eye tracking [UTATE]. The UTATE assesses looking behavior that may reflect visual attention capacities, by using eye-tracking methods. This UTATE was studied in 16 Dutch 18-month-old toddlers. Results showed that the instrument is feasible and generates good quality data. A first indication of sufficient reliability was found for most of the variables. It is concluded that the UTATE can be used in further studies. Further evaluation of the reliability and validity of the instrument in larger samples is worthwhile.

## Introduction

Everyone needs certain attention skills on an everyday basis; to learn about the social and physical context, to accomplish complicated tasks, and to solve problems and adapt to the environment. Attention-related problems hinder daily functioning and could therefore have important negative consequences, such as poor school performance (e.g., Duncan et al., 2007) and social incompetence (e.g., Bennett Murphy et al., 2007). Early detection of attention problems in infancy or at toddler age could result in support and stimulation in order to improve attention capacities (Atkinson and Braddick, 2012). However, standardized and objective measurement tools of attention capacities in early childhood are scarce. In this pilot study we present a detailed description of a newly developed instrument to assess attention capacities in toddlers using eye-tracking methods: the Utrecht Tasks for Attention in Toddlers using Eye Tracking (UTATE).

An important indicator of attention capacities in young children is looking behavior (Colombo, 2002). The challenge, however, is to reliably and accurately assess these looking behaviors and the underlying attention capacities. Previous research often used human observers to assess attention capacities in young children (e.g., Rose et al., 2001, 2009). This method is very time consuming and might result in observer bias (Oakes, 2012). A measurement method that results in more objective and independent data could provide valuable information on attentional processes, especially in a developmental phase when important growth processes may occur. Eye-tracking methods provide an opportunity to get detailed and accurate information on looking behavior in young children (Gredebäck et al., 2010). In addition, the detection rate of eye-movements is quicker, and the relationship between the stimuli presented and the response given, can be checked more precisely. The use of eye tracking techniques might result in better replication of measurements and studies.

With the introduction of the automated corneal-reflection eye-tracker methods, it became possible to use eye-tracking measurements in young children (Aslin and McMurray, 2004). As the quality of the data is dependent on the calibration of the eye-tracking device, specific challenges arise when eye tracking is used with young children. In addition, data may easily become missing due to movements of the child (Oakes, 2012). Nevertheless, this method has been successfully used to assess cognitive development in infants and toddlers. For example by investigating the development of object representations (e.g., Bertenthal et al., 2013), anticipatory looking (e.g., Hunnius and Bekkering, 2010; Paulus et al., 2011) or goal-directed gaze shifts (Gredebäck et al., 2009). Eye-tracking methods have also been used to measure attention capacities in infants by studying the development of selective attention or the ability to disengage and shift attention (Butcher et al., 2000; Hunnius et al., 2006; Amso and Johnson, 2008). However, information is still scarce concerning the potential of eye-tracking methods to assess attention capacities in toddlers in particular.

Theoretically, attention can be divided into three attention systems: orienting, alerting, and executive attention (Posner and Petersen, 1990). Although assumed to be interconnected, these systems are also understood to have unique functions. The *orienting system* is responsible for the capacity to start paying attention to a target (Posner and Petersen, 1990). It involves the ability to engage, disengage, and to shift attention focus. Transposed to looking behavior, functioning of the orienting system is often assessed by determining the duration of a look at some stimulus before looking at something else. Another indicator of orienting is whether the child is capable of shifting its gaze between stimuli (Van de Weijer-Bergsma et al., 2008).

The second attention system, the *alerting* or *vigilant system*, concerns the ability to achieve and to maintain a state of alert attention (Posner and Petersen, 1990). In toddlers, functioning of the alerting system has been assessed by measuring the ability to sustain attention, as represented by the total amount of time the child continues to look at the stimuli during an experiment (Van de Weijer-Bergsma et al., 2008). The ability to achieve a state of alertness can be measured by comparing the reaction times to a stimulus in trials in which someone is made alert, for example by a signaling sound, and trials in which no signaling sound is used.

*Executive attention* is the third attention system that can be distinguished theoretically. It is defined as goal-directed, planned attention, and the ability to inhibit behavior (Posner and Petersen, 1990). In contrast to the first two systems, this system is based on internal or voluntary control of attention, instead of exogenous control, which is the case in both the orienting and alerting system (Sheese et al., 2008). For toddlers, no tasks were available to measure executive attention (Van de Weijer-Bergsma et al., 2008). As the dorsolateral prefrontal cortex is involved in executive functions, tasks that measure functioning of this brain area, such as the delayed response task, were used as indirect measures of functioning of the executive attention system in infants (Van de Weijer-Bergsma et al., 2008).

No studies or assessment instruments were available that examined functioning of the three attention systems simultaneously in children under 3 years of age. Therefore, a test battery that collects objective and standardized data is needed. In the current study, a new test battery of four eye-tracking tasks was developed to assess attention capacities in toddlers: the Utrecht Tasks for Attention in Toddlers using Eye tracking (UTATE). Four existing tasks focusing on attention capacities by observing children's looking behavior, were adapted for use with eye-tracking methods. In addition, the tasks were adapted for use with 18-month-old toddlers. The tasks underlying this measurement were based on tasks used in experimental studies with young infants, like the disengagement task used by Butcher et al. (2000) or a face task that has been used to study information processing (e.g., Rose et al., 2001, 2009). The aim of this pilot study was to describe the four tasks and the potential outcome measures in detail. It is evaluated whether the UTATE indeed is feasible for use with 18-month-old toddlers. In addition, the quality of the data is studied by evaluating the amount of variable position error (i.e., noise) during fixations in relation to the size of the stimuli, which might be a problem when using eye tracking (Holmqvist et al., 2011). Finally, it is evaluated whether the eye-tracker measures indeed show individual variation in the children's looking behaviors during the tasks. Only if these goals would be attained, further studies with the UTATE to first assess its validity and reliability and later to perform actual studies that focus on attention capacities of toddlers, were considered to be worthwhile.

## Methods

### Participants

The sample consisted of 16 Dutch 17- and 18-month-old children, *M* = 17.63, *SD* = 0.50, 50% boys. The children were born full term (i.e., 37–42 weeks) with a birth weight >2500 g. Parents and children were recruited via the hospitals where the infants were born.

The medical ethical committee of the Utrecht Medical Center approved this study as part of a larger study on visual attention capacities of young children. Informed consent was given by the parents. The children received a small gift after the visit and travel expenses of the parents were refunded.

### Apparatus

The Tobii T60 Eye Tracker with an integrated 17-inch TFT screen was used, with a resolution of 1280 by 1024 pixels (Tobii Technology, Stockholm, Sweden). E-prime 2.0 software (Psychology Software Tools, Pittsburgh, PA) was used to present the stimuli on the screen.

### Procedure

**Figure 1:**
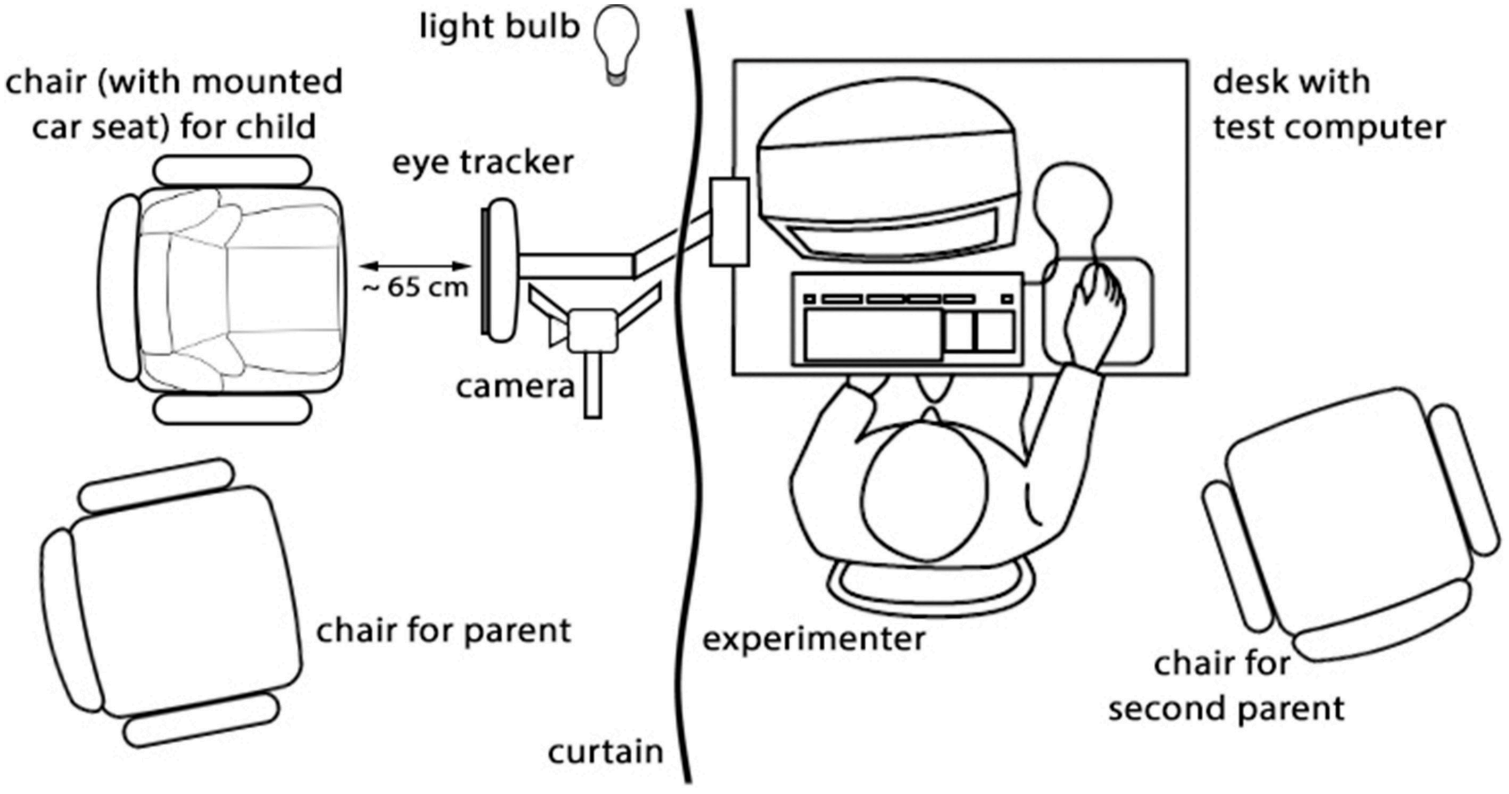
**Visualization of the lab situation**.

The procedure took place in a small, almost dark, and sound-proofed room. See Figure 1 for a visualization of the setup. To make the room less dark (and so less frightening to the children) without distorting the eye-tracking measures, a light bulb was oriented toward the ceiling. The children were placed into a car seat in order to keep them in a sitting position and somewhat constrain them in their movements. The car seat was positioned at a distance of approximately 65 cm from the eye tracker. One of the parents was sitting next to the child and a little to the back, for safety reasons (i.e., to prevent the child climbing out of the chair) and to make the child feel more at ease in the experimental setting. If the child refused to sit in the car seat before or during the experiment, the child was placed on the parent's lap. The test computer, from which the experiments were started, was placed on a desk behind a curtain to prevent the child from seeing the examiner. If more than one parent was present, the second parent was seated next to the experimenter, behind the curtain. The face of the children was recorded with a video camera behind the eye tracker to be able to check the behavior of the child during the procedure.

A nine-point calibration was used, in which a movie clip of a bouncing ball accompanied by sound was presented at nine different points on the screen (i.e., left, middle, and right at the top, center, and bottom of the screen; Hunnius and Bekkering, 2010). Calibration was accepted when the child looked at seven or more of the calibration points. Otherwise several points were recalibrated. After calibration, four tasks were presented in the following fixed order: (1) disengagement task, (2) face task, (3) alerting task, and (4) delayed response task. The whole procedure took about 18 min to complete.

At the beginning of the procedure, before starting the calibration, the parent was told that the procedure included four different tasks in which several pictures were shown, sometimes accompanied by sound. The parent was told to be quiet, unless the child asked for a verbal response. Next to that, the parent was instructed not to direct the attention of the child to the screen when the child looked away. The child was not verbally instructed beforehand.

### Eye-tracker tasks

**Figure 2:**
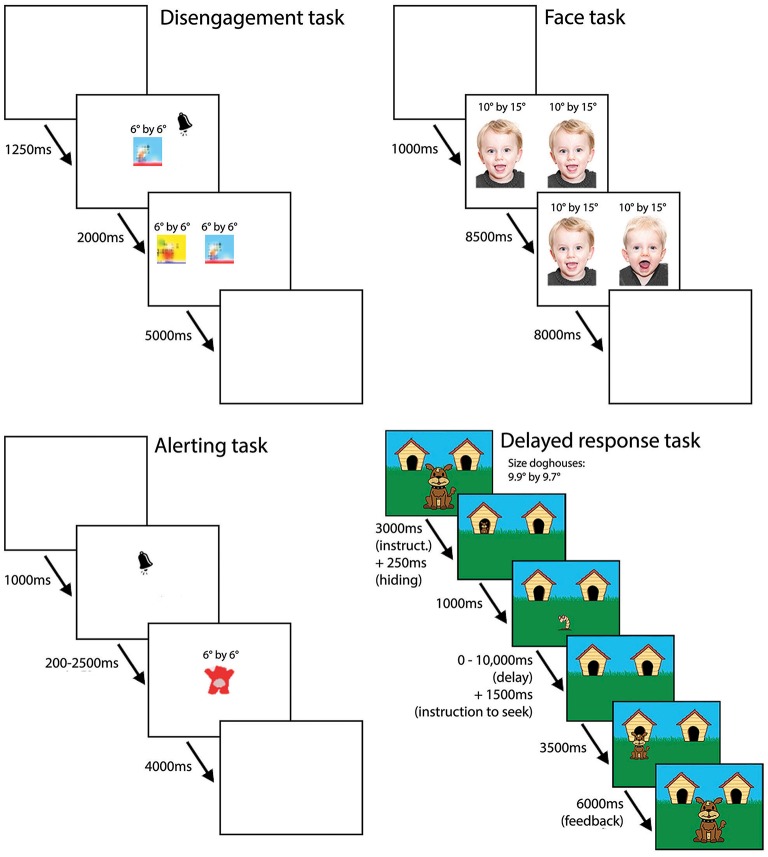
**Visualization of timing and size of the stimuli in the different tasks**.

A visualization of the four tasks is shown in Figure 2. The stimuli used in the tasks can be requested by the corresponding author.

#### Disengagement task

This task is an adaptation of the disengagement task described by Butcher et al. (2000). Stimuli were colorful pictures with a size of 6° by 6°. First, one stimulus was presented at the center of the screen accompanied by a signaling sound to attract the child's attention (i.e., first phase). After 2000 ms a second stimulus appeared on the screen either on the left or right side from the central stimulus with a distance of 3.8° between the stimuli (i.e., second phase). After 5000 ms both stimuli disappeared and after an inter-trial interval [ITI] of 1250 ms the next trial started. The task consisted of 20 trials in which the position of the peripheral stimuli was assigned randomly (half of the time at the left and half of the time at the right side from the central stimulus). The areas of interest [AOI] are the central picture (size of 6° by 6°) during the first phase and the central and peripheral picture (both a size of 6° by 6°) during the second phase.

Five outcome measures from the disengagement task were intended to measure functioning of both the orienting and alerting system. Functioning of the orienting system was intended to be measured by: (1) *mean dwell time*, (2) *transition rate*, (3) *proportion of correct refixations*, and (4) *latency*. The *mean dwell time* is the average duration of the dwells per child averaged across participants. Duration of a dwell (i.e., *dwell time*) is the sum of all fixation durations during one visit in an AIO, as defined by the researcher, from entry to exit (Holmqvist et al., 2011). *Mean dwell time* includes dwells during the first and the second phase of the trial at both the central and the peripheral stimuli. *Transition rate* is the number of transitions during the second phase of the trial divided by the *total dwell time* in the second phase of the trial. A *transition* is “the movement from one AOI to another” (Holmqvist et al., 2011). Because the number of transitions is influenced by the total amount of time the children actually looked at the stimuli (i.e., *total dwell time*), which is a measure of functioning of the alerting system, we controlled for the amount of time the child looked at the stimuli and used the *transition rate* as measure. A *correct refixation* means that the participant refixated from the central stimulus to the peripheral stimulus after the peripheral stimulus is presented, which reflects the ability to disengage and correctly orient to a target. *Latency* is the average time between appearance of the peripheral stimulus and fixation on the peripheral stimulus in trials in which the participant correctly refixated. Shorter *latencies* represent faster transitions. If a child did not look at the central stimulus when the peripheral stimulus appeared, this trial was not taken into account for determining the proportion of correct refixations and the average latency. More *correct refixations*, shorter *latencies*, shorter *mean dwell times*, and higher *transition rates* are thought to be indicative of better functioning of the orienting system (Colombo, 2002; Rose et al., 2002).

An additional outcome measure was expected to measure functioning of the alerting system by assessing the amount of sustained attention, which represents maintenance of a state of alertness: *total dwell time*. The *total dwell time* is the sum of the duration of all dwells per child averaged across participants. *Total dwell time* includes dwells during the first and the second phase of the trial on both the central and the peripheral stimuli. Longer *total dwell times* might reflect better sustained attention, hence a better functioning alerting system.

#### Face task

The face task is based on the “Rose task” described by Rose et al. (2001, 2009). Stimuli were pictures of children's faces (16 different faces presented in 8 fixed sets) with a size of 10° by 15°. First, two identical stimuli (i.e., faces) were presented concurrently for 8500 ms with a distance of 5.5° between the stimuli (i.e., familiarization phase). Next, one of the stimuli changed into a new stimulus (i.e., test phase). Both stimuli stayed on the screen for another 8000 ms. ITI was 1000 ms. The task consisted of 8 trials and the position of the new stimulus was randomly assigned (half of the time at the left and half of the time at the right side of the screen). The areas of interest [AOI] are the two pictures of child faces (both a size of 10° by 15°) during both the familiarization and test phase.

In the face task, three outcome measures were presumed to measure functioning of both the orienting and alerting system. Functioning of the orienting system was intended to be measured by: (1) *mean dwell time*, and (2) *transition rate*. Shorter *mean dwell times* and higher *transition rates* might be indicative of better functioning of the orienting system (Colombo, 2002; Rose et al., 2002). *Mean dwell time* includes dwells at both stimuli during both the familiarization and the test phase. *Transition rate* is based on the transitions and the total dwell time during both the familiarization and the test phase.

One variable was intended to indicate the amount of sustained attention: *total dwell time*. *Total dwell time* includes dwells at both stimuli during both the familiarization and the test phase. Longer *total dwell time* might reflect better sustained attention.

#### Alerting task

The alerting task is an adaptation of the alerting task described by Berger et al. (2000). The response type of this task was changed from touching behavior into looking behavior for use with the eye tracker. The stimulus was a picture of a bear appearing in one of eight different colors with a size of 6° by 6°. The stimulus was presented at the center of the screen for 4000 ms, and the ITI was 1000 ms. The experiment consisted of eight different trial types, which each appeared four times, leading to a total of 32 trials. Two variables varied between trial types: (1) a warning signal (i.e., a ringing sound) preceding the appearance of the stimulus or not (signal and no-signal trials); (2) the interval between the warning signal (or start of trial in no-signal trials) and appearance of the stimulus (200, 500, 1000, or 2500 ms). During the warning signal (or silence in the no-signal trials; duration in both cases 1000 ms) and the interval (200–2500 ms), the screen was white. First, to familiarize the child with the task, four practice trials were administered in which a signal preceded the appearance of the stimulus, and the stimulus followed after 200 ms. Next, 32 trials were administered in semi-random order: four series of the eight different trial types were presented in which the order of trial types within the series was randomly assigned. The eight colors of the bears were randomly assigned, but the same color could not appear in two consecutive trials. The area of interest [AOI] is the picture of the bear (size of 6° by 6°).

In the alerting task, the *difference between latencies* in the no-signal and signal trials was intended to measure of functioning of the alerting system. *Latency* is the average time between appearance of the stimulus and fixation on this stimulus. Larger *differences between latencies* in the no-signal and signal trials, with longer latencies in no-signal trials than in signal trials, are presumed to be indicative of better functioning of the alerting system. Another measure intended to measure functioning of the alerting system is *total dwell time*. *Total dwell time* includes dwells at the stimulus during the presentation of the stimulus. Longer *total dwell times* might reflect better sustained attention.

#### Delayed response task

The delayed response task is an adapted version of the task described by Diamond and Doar (1989). First, a dog and two doghouses were presented respectively at the center, the left top side, and right top side of the screen. The dog houses had a size of 9.9° by 9.7° and the distance between the dog houses was 5.5°. Before the first trial, an introduction was given during which a voice-over told the child that the dog wants to play hide-and-seek: “*Zie je dit hondje? Hij wil verstoppertje met je spelen. Doe je met hem mee?*” (i.e., “*Do you see this dog? He wants to play hide-and-seek with you. Will you play along?*”; duration 6000 ms). At the start of each trial, the voice-over says that the dog is going to hide now: “*Het hondje gaat zich nu verstoppen. Goed opletten!*” (i.e., “*The dog is going to hide now. Pay attention!*”; duration 3000 ms). The dog then moves to one of the two dog houses (250 ms) and disappears after 1000 ms. During the delay, when the dog is no longer visible on the screen as it is hidden in one of the dog houses (varying from 0 to 10 s), a worm pops up in the center of the screen to distract the child from watching the dog houses. In the 0 s delay the worm appearing in the screen is directly accompanied by the voice-over saying “*Waar is het hondje?*” (i.e., “*Where is the dog?*”; duration 1500 ms). With longer delays the worm moves up and down together with a sound, before the voice-over instructs the child to find the dog. After 3500 ms the dog re-appears in the correct dog house and the voice-over tells the child “*Daar is het hondje weer. Hij vindt het een leuk spelletje. Hij wil nog een keertje spelen*” (i.e., “*Here is the dog again. He likes the game. He wants to play again*.”; duration 6000 ms) and then the next trial starts. After the last trial the voice-over tells the child “*Daar is het hondje weer. Hij is nu een beetje moe. Bedankt voor het spelen*.” (i.e., “*Here is the dog again. He is a bit tired now. Thanks for playing*.”). This task consisted of 18 trials. Position of hiding was randomly assigned (half of the time in the left and half of the time in the right dog house, and no more than three consecutive trials in the same position). After three consecutive trials the delay between hiding and the instruction to seek the dog increased from 0 to 10 s with steps of 2 s. The areas of interest [AOI] are the left and right dog house (bot a size of 9.9° by 9.7°) during the period in which the child is searching for the dog and when the dog reappeared.

Functioning of the executive attention system was intended to be measured by: (1) the number of *correct searches* (i.e., the number of trials in which the child looked at the correct dog house directly in response to the voice-over asking where to find the dog), (2) computing the *mean delay* between hiding and the instruction to seek the dog for the trials in which the child looked at the correct dog house. To compute the mean delay, the trials with 0 s delays were excluded, because these trials do not reflect a delay. More *correct searches* and a longer *mean delay* might be indicative of better functioning of the executive attention system.

Furthermore, one other variable was presumed to measure functioning of the alerting system: *total dwell time*. *Total dwell time* includes dwells at the dog houses from the time in the trial that the child is asked to search for the dog until the start of the next trial (total duration per trial 11,000 ms). Longer *total dwell times* might reflect better sustained attention.

### Data analysis

Matlab 7.11 (The MathWorks, Inc.) was used to analyze gaze data. Fixation detection was done by a self-written Matlab program that marked fixations by an adaptive velocity threshold method (Hooge and Camps, 2013). We used an adaptive velocity threshold method to detect fixations because the amount of noise may vary a lot in eye-tracking data (especially with low frequency trackers such as the Tobii T60 and with non-grown-up participants). Many modern saccade and fixation detection methods are partly or fully adaptive to the noise in the data (Smeets and Hooge, 2003; Nyström and Holmqvist, 2010). Velocities were obtained by first fitting a parabola through three subsequent data points. Then we used the derivative of this parabola to estimate the value of the velocity of the second (center) data point. This procedure was repeated for all data points (except the first and the last). In the present analysis, everything that is not a saccade is called a fixation (Holmqvist et al., 2011). To remove the saccades from the signal, we calculated average and standard deviation from the absolute velocity signal. All data points with absolute velocities higher than the average velocity plus 3 times the standard deviation were removed. This procedure was repeated until the velocity threshold converged to a constant value or the number of repetitions reached 25. Then we removed fixations with durations shorter than 50 ms from the analysis. The value of 50 ms was chosen because it is equal to three data samples. When a saccade was removed, the preceding and succeeding fixations were added together. Data of the children were included when they looked at the stimuli at least once during a task, as this provides sufficient information to compute the variables assessed by this task.

The quality of eye-tracking data is reflected by the amount of noise during fixations. By noise we refer to the variable position error that may depend on many factors ranging from eye physiology to calibration method (Nyström et al., 2013). The root mean square (RMS) noise was used in this study. The RMS noise was determined by taking the square root of the sum of the squared angular distances (i.e., distances in degrees of visual angle between subsequent data samples) divided by the number of samples (Holmqvist et al., 2011, p. 35).

To give a first impression of the reliability of the outcome measures, split half reliability was investigated with the Spearman-Brown formula using Pearson correlations between the variables in the odd-numbered trials and the even-number trials.

## Results

### Cooperation of the children

All 16 participants provided data on all four tasks. Therefore, no children needed to be excluded from the analyses. In these 16 children no cases of calibration failure or tracking failure were found and the children were quite compliant with the tasks.

Two children refused to sit in the car seat beforehand and were placed on their parents' lap, after which they participated with all tasks. Three children changed position (i.e., from car seat to parents lap) during the procedure, between the face and alerting task, *n* = 2, or during the alerting task, *n* = 1, because of crying, *n* = 1, or refusal to sit in the car seat, *n* = 2, but they did participate with all tasks. They were not the only children who fussed or showed protest, but in the other cases it was to a lesser extent, so changing positions was not needed for them.

Viewing the video recordings showed that the children generally sat at ease, looked at the screen with interest most of the time, moved a bit with the sounds and sometimes looked at their parents.

### Results of the tasks

In Table 1, means and standard deviations of the 13 variables intended to measure functioning of one of the three attention systems are presented. No outliers (i.e., >3 SD below or above mean) on these variables were found.

**Table 1:** **Descriptive statistics of the outcome measures in all four tasks per attention system**.

	**Mean**	**SD^a^**	**Range**	**25–75% range**	**Possible range**	**Split-half reliability**
**ORIENTING SYSTEM**						
1. DIS mean dwell time (ms)	1453	276	1044 to 2245	1257 to 1594	0 to 140,000	0.74
2. DIS latency (ms)	505	118	356 to 827	438 to 560	0 to 5000	0.33
3. DIS proportion correct	0.97	0.05	0.83 to 1.00	0.96 to 1.00	0 to 1.00	−0.27
4. DIS transition rate^b^	0.49	0.14	0.29 to 0.76	0.37 to 0.57	0 to 8	0.82
5. FACE mean dwell time (ms)	1239	304	689 to 1891	1028 to 1423	0 to 132,000	0.73
6. FACE transition rate^b^	0.65	0.19	0.39 to 1.13	0.52 to 0.73	0 to 8	0.82
**ALERTING SYSTEM**						
7. DIS total dwell time (ms)	93,555	23,453	47,652 to 122,366	71,841 to 115,212	0 to 140,000	0.95
8. FACE total dwell time (ms)	72,055	28,299	14,887 to 101,147	44,150 to 96,427	0 to 132,000	0.91
9. AL total dwell time (ms)	50,017	25,083	7966 to 90,977	29,167 to 69,249	0 to 128,000	0.91
10. AL difference in latency (ms)	136	293	−333 to 611	−132 to 400	−4000 to 4000	−0.04
11. DR total dwell time(ms)	73,469	34,109	10,916 to 140,866	54,050 to 95,784	0 to 198,000	0.94
**EXECUTIVE ATTENTION SYSTEM**						
12. DR correct searches	9.19	3.51	4 to 15	7 to 12.5	0 to 18	0.77
13. DR mean delay (s)	5.39	1.00	4 to 6.67	4.08 to 6.33	0 to 10	0.46

a *Standard deviation between children*.

b *Number of transitions per second*;

*DIS, disengagement task; FACE, face task; AL, alerting task; DR, delayed response task*.

#### Disengagement task

In the disengagement task, the children looked at the stimuli (i.e., both central and peripheral) in 17.50, *SD* = 2.88, out of 20 trials (88%). The average amount of RMS noise is 0.20°, *SD* = 0.09, on the horizontal component of fixation and 0.32°, *SD* = 0.15, on the vertical component, which is respectively 30 and 19 times smaller than the size of the stimuli.

Individual variation was observed in all outcome measures, with less variation seen in the proportion of correct refixations. Most of the children (75%) had a proportion of correct refixations of 1.00, indicating that they refixated correctly in all trials.

#### Face task

In the face task, the children looked at the stimuli in 6.38, *SD* = 1.96, out of 8 trials (80%). The average amount of RMS noise is 0.19°, *SD* = 0.06, on the horizontal component of fixation and 0.28°, *SD* = 0.09, on the vertical component, which is respectively 53 and 54 times smaller than the size of the stimuli. Individual variation was observed in all outcome measures.

#### Alerting task

In the alerting task, the children looked at the stimuli in 19.19, *SD* = 8.03, out of 32 trials (60%). The children looked somewhat more often in the signal trials, *M* = 10.13, *SD* = 4.44, than in the no-signal trials, *M* = 9.06, *SD* = 3.89, *t*_(15)_ = 1.85, *p* = 0.08.

The mean difference in latency between no-signal and signal trials was 136 ms, *SD* = 293, indicating marginally significant shorter latencies in signal than in no-signal trials, *t*_(15)_ = −1.86, *p* = 0.08. In 68.7% of the children, the mean difference in latency had a positive value, showing that the child had shorter latencies in signal than in no- signal trials. The average amount of RMS noise is 0.21°, *SD* = 0.08, on the horizontal component of fixation, and 0.33°, *SD* = 0.13, on the vertical component, which is respectively 29 and 18 times smaller than the size of the stimuli. Individual variation was observed in all outcome measures.

#### Delayed response task

In the delayed response task children needed to be distracted from looking at the dog houses after disappearance of the dog, therefore it was checked whether the distraction (i.e., a worm popping up in the middle of the screen, accompanied by a tune) actually worked. Results showed that none of the children continuously looked at a dog house during the distraction period; they looked at the worm, at the dog houses (but not continuously) or away from both the dog houses and worm. It was concluded that the children indeed were distracted.

The children searched for the dog in 14.13, *SD* = 4.08, out of 18 trials (79%), and they searched correctly in 9.19, *SD* = 3.51, trials. This indicates that, on average, the children searched correctly in 65.6% of the trials in which they searched, which is more than the 50% that would be expected based on chance, *t*_(15)_ = 3.88, *p* = 0.001.

The average amount of RMS noise was 0.14°, *SD* = 0.05, on the horizontal component of fixation and 0.19°, *SD* = 0.08, on the vertical component, which is respectively 71 and 51 times smaller than the size of the stimuli (i.e., the dog houses). Individual variation was observed in all outcome measures.

### Split-half reliability

Split half reliability for each outcome measure is presented in Table 1. A high reliability was found for “total dwell time” in all four tasks, and “transition rate” in both the Disengagement and Face Task. A moderate to high reliability was found for “mean dwell time” in both the Disengagement and Face Task, and “number of correct searches” in the Delayed Response Task. For “latency” and “proportion of correct refixation” in the Disengagement Task, “latency difference” in the Alerting Task, and “mean delay” in the Delayed Response Task, the split half reliability was weak.

### Correlations between the measures

The correlations between the measures are shown in Table 2. Given the small sample size (*n* = 16), we focus on the strength of the correlations, rather than the statistical significance. As can be seen in Table 2, the correlations between measures of the orienting system were overall moderate to strong (ranging from *r* = −0.28 to *r* = −0.87). Only for “latency” in the disengagement task, 2 out of 4 correlations were weak. Also correlations between the measures of the alerting system were mostly moderate to strong (ranging from *r* = 0.20 to *r* = 0.69). Exceptions were found for “total dwell time” in the delayed response task, for which only 1 out of 4 correlations was moderate. Regarding the executive attention system, the two measures were strongly correlated (*r* = 0.77). Due to the small sample size, only strong correlations were significant.

**Table 2:** **Correlations between the outcome measures**.

	**1**	**2**	**3**	**4**	**5**	**6**	**7**	**8**	**9**	**10**	**11**	**12**	**13**
**ORIENTING SYSTEM**													
1 DIS mean dwell time	1												
2 DIS latency	0.49	1											
3 DIS proportion correct	0.55^*^	−0.16	1										
4 DIS transition rate	−0.73^**^	−0.07	−0.44	1									
5 FACE mean dwell time	0.76^**^	0.39	0.28	−0.73^**^	1								
6 FACE transition rate	−0.63^**^	−0.41	−0.29	0.72^**^	−0.87^**^	1							
**ALERTING SYSTEM**													
7 DIS total dwell time	0.63^**^	0.19	0.39	−0.38	0.42	−0.29	1						
8 FACE total dwell time	0.36	0.03	0.27	−0.33	0.63^**^	−0.39	0.46	1					
9 AL total dwell time	0.41	0.06	0.27	−0.20	0.43	−0.17	0.31	0.45	1				
10 AL latency difference	−0.13	−0.03	−0.16	0.17	0.30	−0.10	0.20	0.69^**^	0.23	1			
11 DR total dwell time	0.08	−0.13	0.22	−0.34	0.18	−0.29	0.10	0.38	0.10	0.11	1		
**EXECUTIVE ATTENTION SYSTEM**													
12 DR correct searches	0.07	−0.16	0.26	−0.26	0.12	−0.12	0.05	0.49	0.10	0.18	0.82^**^	1	
13 DR mean delay	0.15	0.18	0.25	−0.18	0.30	−0.37	0.15	0.61^*^	0.19	0.23	0.69^**^	0.77^**^	1

* *p < 0.05*,

** *p < 0.01*;

*DIS, disengagement task; FACE, face task; AL, alerting task; DR, delayed response task*.

## Discussion

In this paper, the Utrecht Tasks for Attention in Toddlers using Eye tracking (UTATE) is described in detail, and its potential to study attention capacities in 17- and 18-month-old toddlers is evaluated. Regarding the feasibility of the eye-tracking procedure for toddlers, it was found that the children cooperated quite well. Data were available from all participants on all four tasks. The quality of the data was good; the amount of RMS noise was much smaller than the size of the stimuli and was smaller than the precision reported by Tobii (2011). Individual differences were observed in most outcome measures. Consequently, it was concluded that the UTATE has the potential to elucidate important variation in looking behavior. In addition, a first indication of sufficient reliability was found for most of the variables.

Three of the four tasks (i.e., disengagement task, face task and delayed response task) were interesting enough for the children to participate in most of the trials. For the alerting task, the looking rate was somewhat lower, i.e., 60%. This task may require more effort of the children's attention capacities, because it came later in order, has many trials and therefore lasts long, and the same stimulus (the same bear appearing only in a different color) was used each time. Exactly for this reason, however, this task may provide valuable information regarding individual variation between the children in sustaining attention.

Individual variation in looking behavior was found in most outcome measures, with less variation seen in the proportion of correct refixations in the disengagement task. Most of the children (i.e., 75%) correctly refixated in all of the trials. Although this measure differentiated between the performances of infants until 6 months of age in a previous study (Butcher et al., 2000), this was not the case for the 17- and 18-month-old toddlers in our study. Perhaps, the capacity to refixate correctly is already fully developed at this age and therefore no longer differs as much between individual children. The children that were unable to refixate in all of the trials, however, may have difficulties in attention regulation strategies. Further research might focus on intra-individual differences within and between tasks to study individual patterns of attention capacities.

Good split-half reliability was found for nine out of 13 variables. Weak reliability was found for latency and proportion of correct refixations in the disengagement task, latency difference in the alerting task, and mean delay in the delayed response task. Low reliability of the proportion of correct refixations might be explained by the small variation in this variable. For mean delay in the delayed response task, low reliability might be due to differences in the delay per trial. As the delay increases with 2 s for every three consecutive trials, it was difficult to make an appropriate split, so other measures of reliability, such as test-retest reliability, are needed to study reliability of this variable. As yet we have no clear explanation for the low reliability of latency in the disengagement task and latency difference in the alerting task. As this study included only 16 children, further evaluation in a larger sample is needed. Next to that, future research should investigate the test-retest reliability of the measures.

The correlations between the outcome measures gave a first indication that the different constructs of attention may be measured, because most of the correlations between variables that were expected to measure functioning of the same attention system were moderate to strong. In other words, there are first indications that children who scored low (or high) on one measure of a specific attention system were also more likely to score low (or high) on other measures of that same system. However, because of the small sample size we have to be cautious, and further research with these tasks using a larger sample is needed.

As no other studies focusing on attention capacities of toddlers using eye tracking were found, no comparison is made to results of others. In addition a comparison would always be difficult in view of differences in design and stimuli used.

This study provided a preliminary evaluation of the potential of the UTATE in a small number of children. We conclude that it is worthwhile to conduct further studies with the UTATE because it resulted in good quality data and it is feasible for use in studies on attention capacities in toddlers. The reliability and validity of the instrument need to be studied further in larger samples. This report also intended to describe the UTATE in great detail to allow replication and use of the UTATE by other researchers. Currently validation studies with a larger sample are being conducted to investigate whether the supposed underlying attention systems (i.e., orienting, alerting, and executive attention) are indeed measured with these tasks (De Jong et al., 2016). In addition, it is studied how the results of the UTATE compare to other measures of attention, as well as to more general assessments of developmental level of toddlers. Finally, it will be studied whether the UTATE differentiates between children at high or low risk for developing attention and developmental difficulties. If the UTATE is able to do so, the battery could be used in studies on early attention development, on individual trajectories of attention development and in studies aimed at developing interventions to support high risk children.

## Author contributions

MDJ designed the study, performed the data collection, carried out the analyses, drafted the initial manuscript, revised the manuscript and approved the final manuscript as submitted. MV designed the study, reviewed and revised the manuscript and approved the final manuscript as submitted. IH prepared the eye-tracking data for analyses, assisted in data analyses, reviewed and revised the manuscript and approved the final manuscript as submitted. AVB conceptualized and designed the study, reviewed and revised the manuscript and approved the final manuscript as submitted.

### Conflict of interest statement

The authors declare that the research was conducted in the absence of any commercial or financial relationships that could be construed as a potential conflict of interest.
